# Scintigraphic Demonstration of Urine Extravasation Secondary to Acute Ureteral Obstruction: A Case Report and Some Considerations about Acute Ureteral Obstruction

**DOI:** 10.1100/tsw.2006.389

**Published:** 2006-10-09

**Authors:** Federico M. Sarmiento, Arístides J.H. Sarmiento, Edgardo Bardoneschi, Arístides H. Sarmiento

**Affiliations:** ^1^ Servicio de Medicina Nuclear, Hospital Militar Central, Luis M Campos, Buenos Aires, Argentina; ^2^ Servicio de Urología del Hospital Militar Central Cir. Ej. Dr. Cosme Argerich, Luis M Campos, Buenos Aires, Argentina

**Keywords:** urinary lithiasis, flank pain, renal colic, acute ureteric obstruction, spontaneous urine extravasation, renal scintigraphy, diagnosis

## Abstract

Acute ureteral obstruction produces renal damage and complications that are proportional to the severity and length of the obstruction. Anatomic diagnosis of the obstruction may be insufficient to manage the patient. Intravenous urogram (IVU) is the method usually advised by radiologists to obtain functional information, but requires iodinated contrast agents. IVU anatomic information is superior to anatomic information obtained with renal scintigraphy, but normally the physician already has the anatomic information (unenhanced CT or ultrasound). A renal scan offers better physiologic information than the IVU, has neither adverse effects nor complications, is accurate to confirm or discard significant ureteral obstruction, and depicts obstruction complications. This paper presents a patient with spontaneous urine extravasation secondary to acute renal obstruction who is diagnosed with renal scintigraphy. The authors describe the scintigraphic signs of extraperitoneal, diffuse perinephric, urine extravasation and emphasize the role of renal scintigraphy in diagnosis and follow-up of renal colic.

## INTRODUCTION

The use of intravenous (IV) contrast media in the emergency room is limited, in some countries, by the need for lab work (serum protidogram) to reduce the risk of adverse events. This is why unenhanced CT scans are preferred over intravenous urograms (IVUs) and enhanced CT scans in such a setting. As long as the amount of upper urinary tract dilatation is not related to the severity of the obstruction, renal scintigraphy could be proposed as the standard to evaluate the degree of obstruction in the emergency room in selected cases.

Urine extravasation secondary to acute ureteral obstruction is an unusual complication. The authors demonstrate this complication with technetium99m-diethylenetriaminopentaacetic acid (99mTc-DTPA) renal scintigraphy, describe some limitations of anatomic diagnostic methods (unenhanced CT and ultrasonography [US]), and emphasize the indication of renal scintigraphy over IVU in the functional evaluation of acute ureteral obstruction. The scintigraphic signs of urine perinephric extravasation and the follow-up after the decompression procedure are described.

Renal sonography is the initial technique most used to evaluate patients with flank pain because of its low cost, accessibility, differential diagnosis with other pathologies, and lack of irradiation[1,2]. Renal sonography has known limitations and unenhanced CT has been proposed as the best screening method to study the patients with suspected renal colic[3,4,5,6,7]. The information obtained with unenhanced CT and sonography is only anatomic. Anatomic information may be sufficient in some clinical situations, but in many others, the information about renal functional repercussion is essential.

IVU is the method that is usually indicated to complement the anatomic information[13], but requires the use of iodinated contrast agents. There are well-known reactions that iodinated contrast agents may produce: hives, facial or laryngeal angioedema, bronchospasm, pulmonary edema, hypotension, arrhythmias, and renal function deterioration. Diabetes, renal insufficiency, congestive heart failure, multiple myeloma, and hyperuricemia are concomitant pathologies with increased risk of contrast-related nephropathy.

Renal scintigraphy with 99mTC-mercaptoacetyltriglicine (99mtc-MAG3) are confirmed methods to evaluate the functional significance of ureteral obstruction. They are specific studies directed to nephroureteral evaluation, and this may be their major limitation because a significant number of patients with flank pain do not have urologic pathologies and, in these patients, CT or US may be initially more useful[8,9,10,11,12].

99mTc-DTPA extraction is proportional to glomerular filtrate whereas 99mTc-MAG3 represents renal plasma flow. The uptake and excretion of 99mTc-MAG3 is faster than 99mTc-DTPA and it was proposed as the better radiotracer in renal insufficiency. However, renal scintigraphy with 99mTc-DTPA is cheaper than 99mTc-MAG3 and has similar diagnostic accuracy, at least in patients with normal renal function or moderate renal insufficiency.

Despite the clinical utility of renal scintigraphy, it is an underused method. In fact, it is not even mentioned in a review on radiologic methods in urologic emergencies.

## CASE PRESENTATION

A 54-year-old male, without nephrourologic history, consulted in the emergency department because of 36 h of severe left flank pain, microhematuria, left abdomen defense with signs of peritoneal inflammation, and 37.5°C temperature.

KUB (Fig. 1) and renal sonography (Fig. 2) did not demonstrate lithiasis, hydronephrosis, or renal abnormalities. Presumptive diagnosis was diverticulitis, pyelonephritis or renal abscess. Unenhanced CT demonstrate perinephric stranding, suggestion of an anterior perinephric collection, slight pelvic and ureteral dilation, and an 8-mm lithiasis localized in the middle third of the left ureter (Figs. 3 and 4).

The CT perinephric fluid collection was interpreted as urine extravasation or inflammatory fluid.

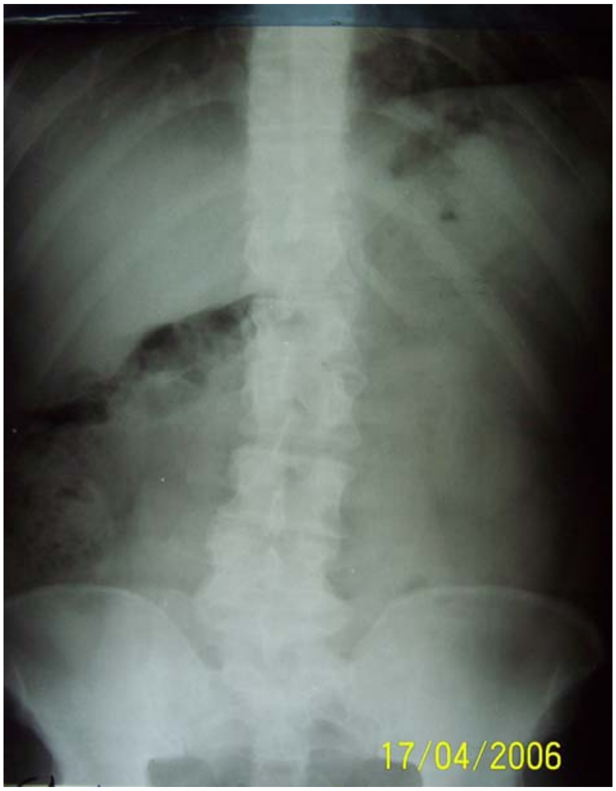

**FIGURE 1.** KUB (4/17/06). There are not evidence of lithiasis.

99mTc-DTPA renal scintigraphy was indicated to evaluate the functional repercussion of the ureteral obstruction and confirm the urine extravasation. The renal scan demonstrated normal perfusion, symmetric renal differential function, no delayed visualization of pyelocalyceal systems, normal right renographic curve, delayed time to peak activity, and delayed excretion in left kidney.

The dynamic sequential views demonstrate a urine extravasation in the superior fornix of the left kidney and perinephric extravasation with an apparent increase kidney size in the 20-min views (Figs. 5–7). Severity and prolonged pain, stone size, and the urine extravasation determined the intervention to decompress the urinary system. A double J catheter was endoscopically introduced (Fig. 8) and the symptoms subsided after 24 h. A renal scan 48 h postprocedure demonstrated normal left kidney excretion and no urine extravasation. It was a small focal upper left kidney defect, probably in the fornix with the tear (Fig. 9).

## DISCUSSION

KUB demonstration of ureteral lithiasis it is not easy; even about 90% of the stones are radiopaque. Sinclair et al.[14], using IVU, detected ureteral lithiasis in only 44/69 patients (64%). This low sensitivity is explained because of the small size of the stone, gas and intestinal feces, low calcium content, totally radiolucent stone (indinavir), or recent spontaneous elimination.

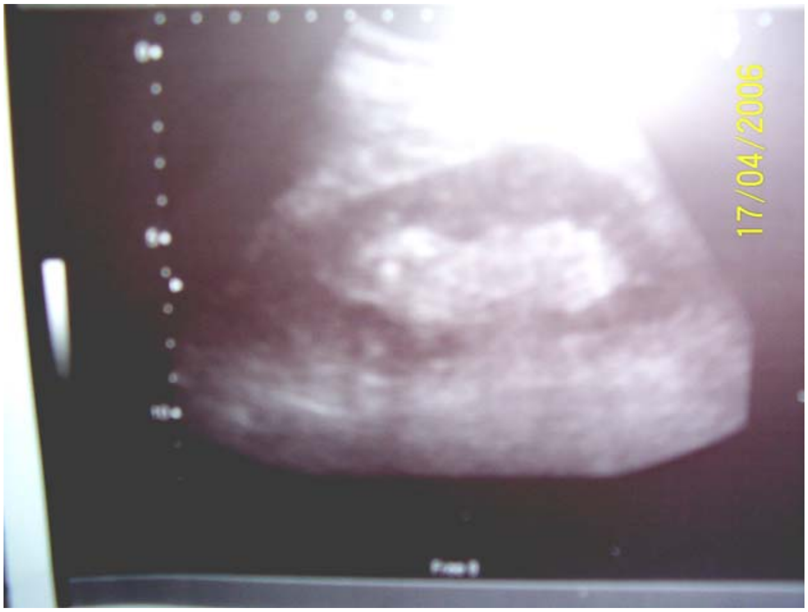

**FIGURE 2.** Left kidney ultrasound (4/17/06). There are not signs of hydronephrosis, lithiasis or urinary extravasation.

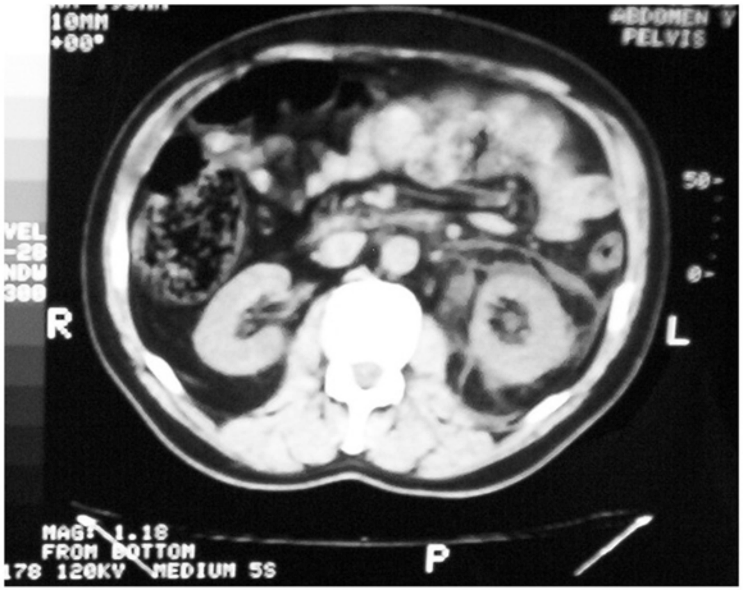

**FIGURE 3**. Unenhanced CT (4/17/06). Perinephric stranding, small perinephric collection and ureteral dilatation.

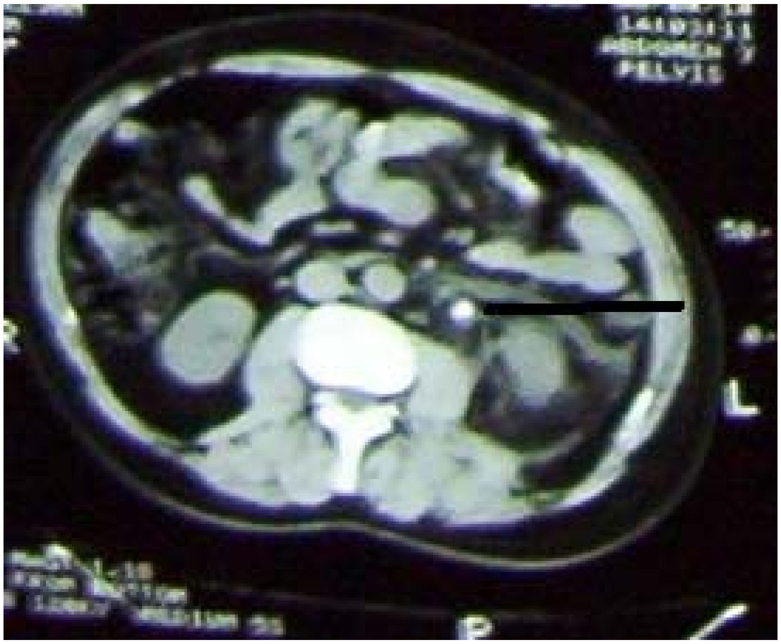

**FIGURE 4.** Unenhanced CT (4/17/06). Ureteral lithiasis (8 mm) (black line)

Renal sonography also has diagnostic limitations. Sonographic demonstration of the stone is possible only when it is localized near the bladder or renal pelvis, but is almost impossible for those localized in the middle third of the ureter. Renal sonography has a sensitivity of only 37% to detect ureteral lithiasis; including hydronephrosis as an obstruction sign, the sensitivity increases to 74%. Hydronephrosis may be minimal or absent in 11–35% of the patients[15].

Dilation of pyelocalyceal system may be present and due to vesicoureteral reflux, previous dilation of the urinary tract, normal variants, parapyelics cysts, hydrocalices, and full bladder. Hydronephrosis may be absent in recent obstructions, intrarrenal pelvis, or pelvis with low compliance, dehydration, or urinary tract decompression because of urine extravasation.

Unenhanced CT has better contrast resolution than simple X-ray and has been proposed as the best screening method to study the patient with acute flank pain. The procedure has no complications, is fast, has good sensitivity to localize stones, and is capable of diagnosing different pathologies with similar characteristics than renal colic.

Size and localization of the stones are important to define treatment; 90% of calculus <4 mm and 50% of those between 5–7 mm are spontaneously eliminated, 8-mm calculus usually needs urologic intervention[16].

Unenhanced CT has several pitfalls. Phleboliths, seminal vesicle calcifications, administered previous oral contrast, treatment with prostatic seeds, calcified adenopathies, and pelvic calcifications may be confused with ureteral lithiasis. Some signs help the differential diagnosis (ureteral rim edema, tail sign, radiolucent center).

Some stones may not be diagnosed in unenhanced CT because they are very small, partial volume effect, low attenuation, totally radiolucent (indinavir), breathing during the acquisition, scarse retroperitoneal fat, or previous elimination.

There are unenhanced CT secondary signs of ureteral obstruction: ureteral or pyelocalyceal dilation, perirenal fat inflammatory changes, unilateral nephromegaly. These secondary obstruction signs may the only diagnostic clue when CT does not demonstrate the stone. Absence of secondary signs or direct demonstration of calcifications along the ureter are usually indirect evidence that there is no ureteral obstruction.

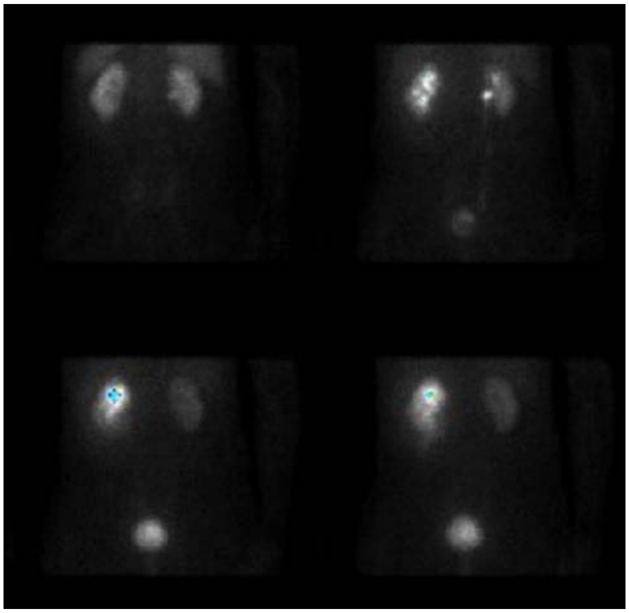

**FIGURE 5.** 99mTc-DTPA renal scintigraphy (4/17/06). Normal right kidney. Focal tracer accumulation in upper left pole and urine extravasation through left superior fornix and perirrenal diffusion.

Presence of secondary signs, without stone, are not specific of ureteral obstruction; perinephric stranding may be seen in ureteral obstruction and pyelonephritis[10,11,15], and urinary tract dilation has same diagnostic limitations other than those described for US.

Unenhanced CT and sonography are purely anatomic methods. They do not offer any information about the compromise of renal function.

Renal scintigraphy is the logical complement of this anatomic information. There are very exceptional situations in that IVU may be preferred to renal scintigraphy.

There are several advantages to the use of renal scintigraphy over IVU: the only patient preparation is oral hydration, it is free of complications, it is well accepted by the patients, the irradiation dose is lower than IVU and CT, and the diagnosis is obtained in 20–30 min.

Renal scintigraphy confirms the ureteral obstruction and its severity, allows the evaluation of renal perfusion, quantifies renal function total and differential, detects urine extravasation, and it is ideal to follow-up the evolution of the patient.

This case is interesting because it demonstrates the diagnostic value of renal scintigraphy in a patient whose initial diagnosis of renal colic was discarded because of normal renal sonography. Clinical manifestations, fever, left flank defense, and peritoneal signs suggested diverticulitis or perirenal abscess.

Unenhanced CT demonstrated ureteral lithiasis and perinephric stranding, but doubts remained about infection. The demonstration of urinary extravasation with the renal scan explained the patient signs and symptoms, and defined the urgent decompression of the urinary tract with resolution of pain and inflammatory signs. A contrast CT may have demonstrated the urine extravasation, but was not ordered because that diagnosis was not suspected initially.

Spontaneous urinary extravasation is a well-known complication of acute ureteral obstruction and had been demonstrated with IVU, contrast enhancement CT, and ultrasound.

Urinary extravasation secondary to acute ureteral obstruction may occur when intrapelvic pressure is higher than 35–40 mm water through pyelosinusal reflux. The fornix may have a tear and through it, the urine infiltrates renal sinus and perinephric space.

Urine extravasation usually occurs in superior or inferior fornices because, in them, the papillaes have a composed morphology and render possible the intrarrenal reflux of urine. Other kidney regions may also have compounded papillaes, but they are more frequent in renal poles[17].

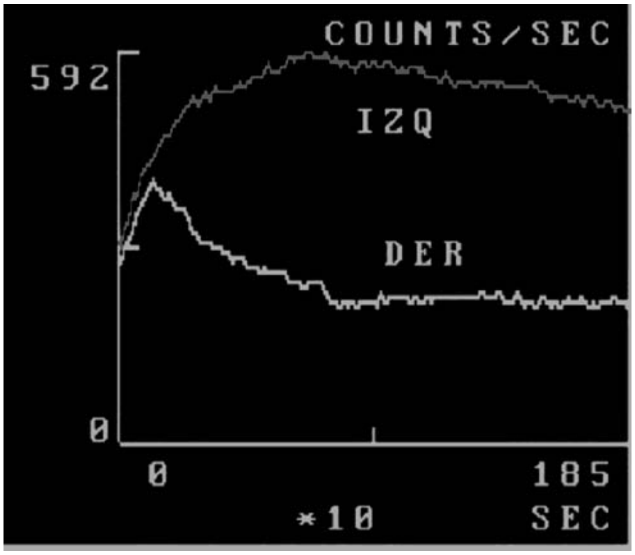

**Figure 6.** Prolonged left nephrogram with delayed peak activity.

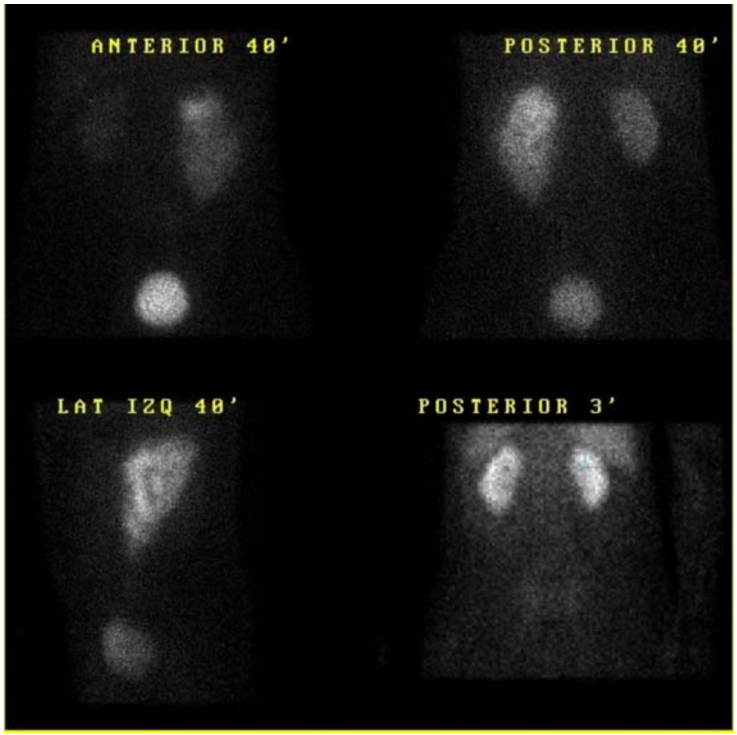

**FIGURE 7.** Static renal views demonstrate the apparent increase left renal size at 40' in comparison with 3' images, due urine perirrenal diffusion.

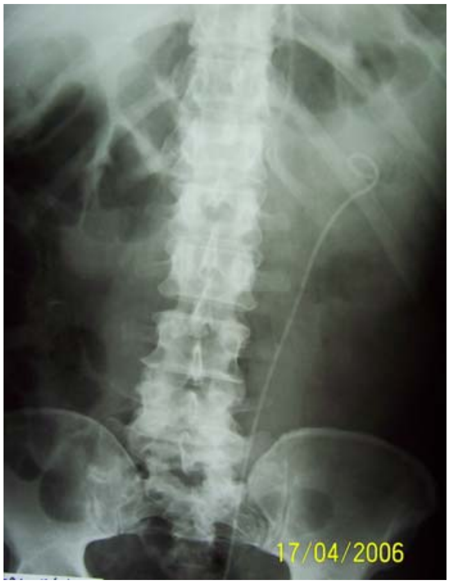

**FIGURE 8.** Double J catéter (7/17/06).

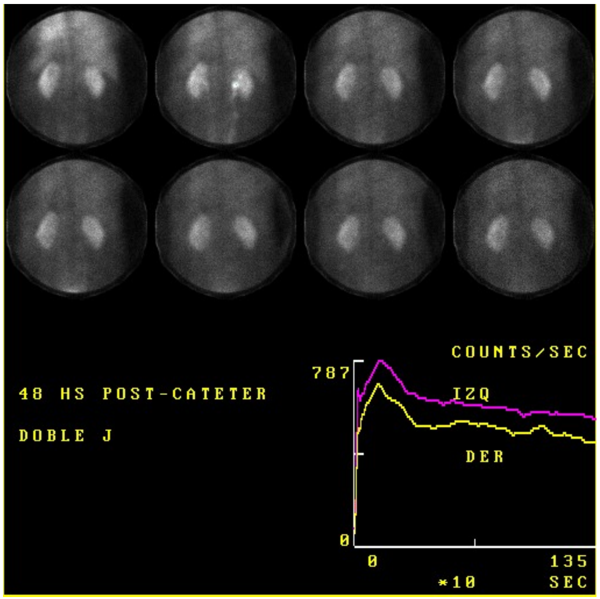

**FIGURE 9.** Renal scintigraphy 48 hs. post-catheter introduction (4/19/06). Normal study.

Urine extravasation in retroperitoneum produces inflammation, severe pain, lipolisis, round cell infiltration, and fibrosis. Urine may extravasate intra- or extraperitoneally.

Extraperitoneal collections may be subcapsular or perinephric. Subcapsular collections are localized between renal parenchyma and renal capsule, and their scintigraphy manifestation would be a persistent focal increase activity in renal cortex.

Perinephric urine extravasations may be diffuse or localized, and their location is between renal capsule and Gerota fascia.

The case presented had an extraperitoneal, perinephric diffuse urine extravasation, secondary to a tear in the superior fornix. The initial extravasation was observed as a collection of activity in the superior fornix that quickly diffused to the perinephric region. The extravasation must not be confused with dilatation of the urinary tract. Urinary tract dilatation secondary to ureteral obstruction affects the renal pelvis and calices, whereas urine extravasation is initially localized and affects one calyx, the others may not be dilated.

The diffuse, perinephric urine extravasation manifests as an apparent nephromegaly in 20- to 30-min views, compared with 5-min views. This is a sign that we are not aware it was described.

Renal scintigraphy in acute ureteral obstruction frequently demonstrates a prolonged nephrographic phase and in the 20- to 30-min views, renal activity is higher than in 5-min views. We should not mistake apparent delayed larger kidney because parenchymal tracer retention with perirrenal diffusion of urine[11].

Sfakianakis et al. and Puri et al. reported urinary extravasation in acute ureteral obstruction detected with renal scintigraphy using 99mTc-MAG3 and 99m-TcDTPA, but they did not show the renal images[12,18].

In our literature revision, we did not find scintigraphic demonstration of this complication. In fact, in our hospital, using renal scintigraphy in the diagnoses and follow-up of renal colic from 1976, this is the first time that we saw it in the renal scan.

We believe that in the study of renal colic, renal scintigraphy is underused. Unenhanced CT and renal US are useful anatomic methods, but in the functional evaluation of the obstruction, the best method is renal scintigraphy and not the IVU.

Renal scintigraphy only requires oral hydration, it is free of complications, and takes no more than 20–30 min. It is useful to determine urologic intervention or medical treatment, and in the follow-up to evaluate renal function damage, complications, persistent obstruction, or spontaneous deobstruction.

Ureteral stones have many clinical and physiologic expressions; from severe pain to almost asymptomatic, from complete obstruction to partial obstruction, from persistent obstructions to intermittent ones, or even stones without evidence of obstruction.

Once the ureteral obstruction is diagnosed, the surgeon must decide how long to wait to deobstruct the urinary tract. In the presence of complete obstruction, renal damage seems to begin in 18–24 h. After 5 days of complete obstruction (and surely after 2 weeks) the renal damage is irreversible. Partial obstruction modifies the mentioned periods, but may also produce irreversible renal damage[19].

This case report is presented to demonstrate the scintigraphic signs of spontaneous urine extravasation secondary to acute ureteral obstruction and the clinical utility of the renal scan. Our clinical conclusions are coincident with those reported previously[12]; renal scintigraphy must be considered in the management of urinary lithiasis when it is necessary quantify the degree of upper urinary tract obstruction or whenever other imaging techniques are not conclusive.

We believe that despite less-anatomic resolution, renal scintigraphy must be indicated in preference to IVU when functional information is necessary because it is not associated with adverse reactions.

One reason that may be suggested in opposition to renal scintigraphy is the cost of the study. In our hospital, we use 99mTc-DTPA, a tracer cheaper than 99mTc-MAG3. The cost of the nuclear study in our hospital is not very different than that for the IVU.

We do not use routinely IV furosemide injection in acute renal obstruction because usually the basal renal scan is diagnostic[9,10,11]. Hyperosmolar contrast agents and furosemide at 0 time may produce a sudden increase of urinary tract pressure and potentially demonstrate more frequent urine extravasation than basal renal scan, but probably that kind of forced extravasation will not have same prognostic significance than the spontaneous one.

99mTc-MAG3 probably would have advantages over 99mTc-DTPA in the diagnosis of renal colic in patients with renal insufficiency, but we do not have experience about it.
